# Unraveling the Impact of Adsorbed Molecules on Photocatalytic Processes: Advancements in Understanding Facet-Controlled Semiconductor Photocatalysts

**DOI:** 10.3390/molecules29102290

**Published:** 2024-05-13

**Authors:** Anna Kusior, Kinga Michalec, Anna Micek-Ilnicka, Marta Radecka

**Affiliations:** 1 Faculty of Materials Science and Ceramics, AGH University of Krakow, al. Mickiewicza 30, 30-059 Krakow, Poland; kmichalec@agh.edu.pl (K.M.); radecka@agh.edu.pl (M.R.); 2 Jerzy Haber Institute of Catalysis and Surface Chemistry, Polish Academy of Sciences, ul. Niezapominajek 8, 30-239 Krakow, Poland; anna.micek-ilnicka@ikifp.edu.pl

**Keywords:** Fe_2_O_3_, nanocrystals, photocatalysis, band gap, adsorption/desorption

## Abstract

This work aims to demonstrate that the Fe_2_O_3_ nanocrystals’ adsorptive and photocatalytic properties can be adjusted by exposing the crystal facets that are functionalized. To this end, cube- and disc-like structures were synthesized using a metal ion-mediated hydrothermal route. Thereafter, some of the samples were annealed at 500 °C for 3 h. Our paper combines the experimental part with theoretical calculations of the obtained materials’ band edge positions. The results reveal that—aside from hematite—the as-synthesized discs also contain γ-FeOOH and β-Fe_2_O_3_ phases, which transform into α-Fe_2_O_3_ during annealing. The hydrodynamic diameter, zeta potential, and adsorption kinetics measurements show that the cube-like samples exhibit the highest affinity for cationic, whereas the discs have an affinity for anionic dye. Measurements of the wall zeta potential also reveal that annealing the discs modifies their surface state and ability to adsorb molecules. Photocatalytic tests show that the as-synthesized powders have better photocatalytic performance toward methylene blue decomposition than the annealed ones. The observed small changes in the concentration of the MO during illumination result from the energy band structure of the cube-like crystal orientation.

## 1. Introduction

Photocatalytic processes are one of the leading directions toward pollutant removal [1,2]. It is well known that the overall process of a semiconductor’s photoreaction is focused on the generation of electron–hole pairs via the absorption of energy greater than or equal to its bandgap [3,4]. As a consequence, photogenerated electrons and holes move through the bulk to the surface, where they drive redox reactions [5].

In the literature, many semiconductors have been proposed for photocatalytic applications, including metal oxides, metal chalcogenides, metal nitrides, carbon nitrides, and III–V compounds [5,6]. TiO_2_, ZnO, Fe_2_O_3_, BiVO_4_, and CdS are the typical photoactive materials [1,7]. Unfortunately, there are no semiconductors that fulfill all the requirements for photocatalysts [8]. The methods that may be applied to improve the efficiency of semiconducting materials in a photodegradation process include (1) doping or co-doping with aliovalent ions; (2) the incorporation of noble metals; (3) the creation of composite materials, plasmonic metal–semiconductor composite photocatalysts, dye-sensitized semiconductors, semiconductor–semiconductor heterojunctions, multiphase heterojunctions of a semiconductor, semiconductor–carbon composites; (4) and the modification of microstructure and morphology [6,9,10,11,12,13,14,15].

However, to fully understand the mechanisms that underlie photodegradation, adsorption processes should be taken into account since they provide data of fundamental importance for the design of improved photocatalysts.

Henderson [16] proposed seven key factors that should be considered when describing the fundamental processes important to photocatalysis: (1) photon absorption, (2) charge transport and trapping, (3) charge transfer, (4) molecular adsorption, (5) reaction mechanism, (6) poisons and promotors, and (7) the surface and structure of a material. These factors determine the characteristics of a heterogeneous photocatalytic reaction, which involves two steps. Firstly, dye molecules are adsorbed on the photocatalyst’s surface. Secondly, the dye starts to undergo photodegradation. The overall efficiency of a photocatalytic system is therefore strictly dependent on the proper selection of the parameters responsible for both the adsorption and photodecomposition of the dye. The adsorption capacity of a given semiconductor toward reactants plays a crucial role in its photocatalytic performance [4,17,18]. On the one hand, the initiation of photodecomposition is related to the adsorption of the dye via electrostatic interaction, which depends on the nature of a dye as well as the surface and bulk properties of a semiconductor. The selection of appropriate conditions for the adsorption processes to occur is likewise important [4,19,20]. On the other hand, photo-induced electrons and holes can react with the adsorbed oxygen, water, and surface hydroxyl groups to create reactive oxygen species (ROS) [21,22]. ROS, the most important of which are the superoxide anion radical, hydrogen peroxide, singlet oxygen, and hydroxyl radical, can react with dyes, decomposing them [22].

Over the past decades, significant progress has been made in the field of materials development. It is believed that the positive role of defects in enhancing adsorption performance can be recognized. The new approach to sorption processes by the control of defects will elucidate defect engineering’s function in enhancing each step of adsorption from the fundamental mechanism point of view. Our early studies of nanocrystals showed that regulating exposed crystal facets could improve their photocatalytic properties [23,24]. In this work, we have undertaken studies to determine the effect of annealing on the structural and adsorptive properties of the cube- and disc-like Fe_2_O_3_ nanocrystals toward cationic and anionic dyes. The aim was to demonstrate that the Fe_2_O_3_ nanocrystals’ photoactivity can be adjusted by exposing modified crystal facets. For this purpose, commercial powder with an irregular shape was used as a reference material. A model based on the crystal orientation of the particles, which affects the energy band structure, was applied to correlate the adsorption/desorption process and photocatalytic properties.

## 2. Results and Discussion

### 2.1. Photocatalysts Characterization

**Figure 1:**
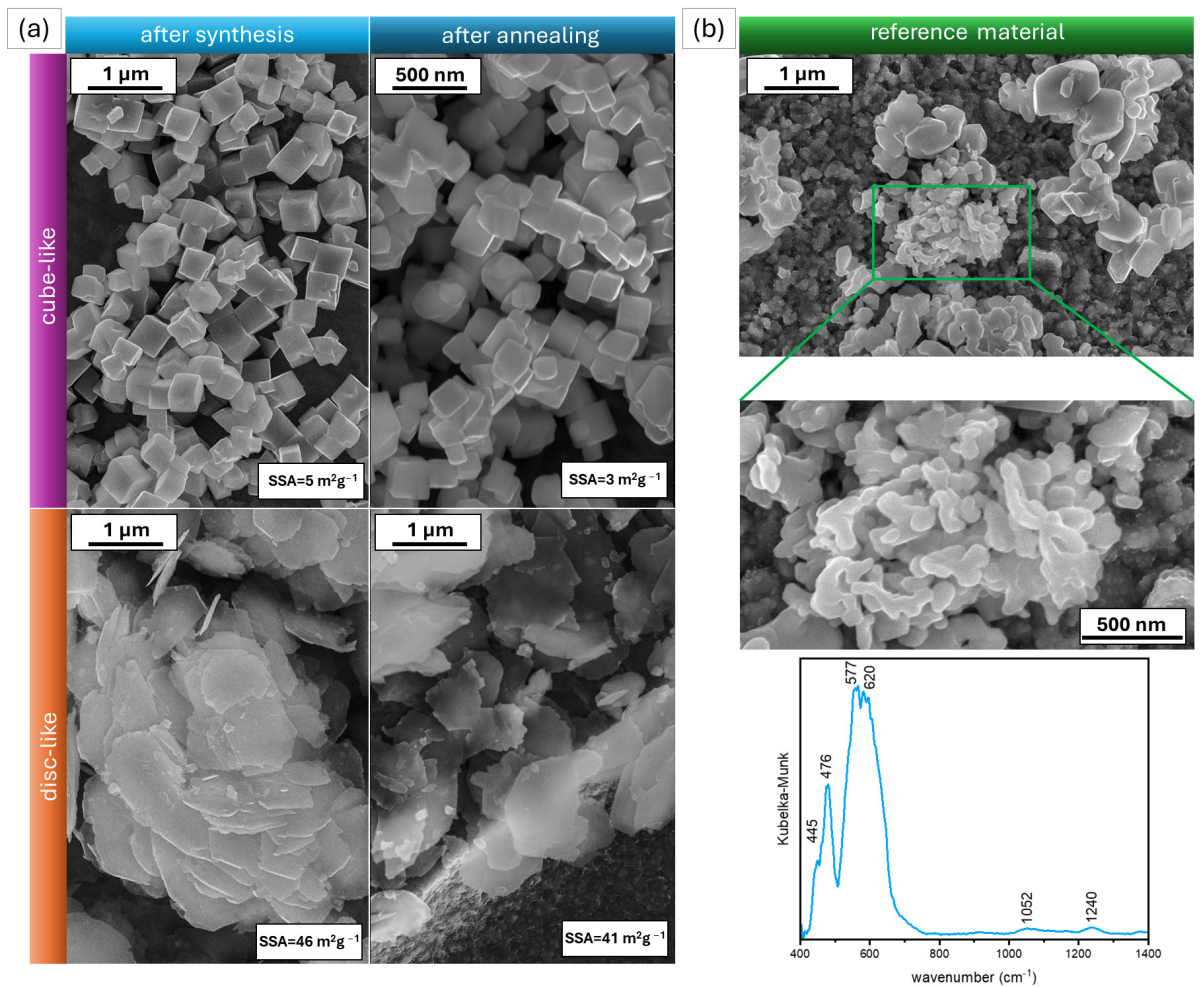
(**a**) SEM images of the obtained iron oxide-based materials in the form of cubes, and discs with measured specific surface area (SSA). (**b**) Microstructure analysis of the unshaped reference powder with FTIR spectra.

Figure 1 shows the as-synthesized and annealed samples. Through the selective adsorption of Zn^2+^ and Al^3+^ ions in specific facets, it was possible to obtain cube and disc-like particles, respectively. The IC powder consists of cubes with an edge length of about 405 nm (with exposed surfaces (014), (114) and (104)) [23]. The disc-like materials (ID) had a hexagon-like surface with a diameter of about 1255 nm and a thickness of around 141 nm (exposed facets (110) and (2–10)). The shape of the sample was not affected by annealing. However, both IC-HT and ID-HT are characterized by slightly larger grain sizes, of about 472 nm and 1410 nmx182 nm, respectively. In addition to microstructural differences, the powders describe different surface properties. The cubes are characterized by a low specific surface area (5 m^2^g^−1^ for IC and 3 m^2^g^−1^ for IC-HT) in contrast to disc-like particles, while the measured SSA exceeds more than 40 m^2^g^−1^ (Figure 1).

The commercial powder is composed of variously shaped and sized particles. The determined d_SEM_ of the particles ranges from 238 nm to 875 nm (Figure 1). As a reference material, the powder should be α-Fe_2_O_3_. The Fourier transform infrared (FTIR) spectra confirm the hematite structure. Visible bands at about 445, 476, 577, and 620 cm^−1^ may be attributed to Fe-O vibrations [25,26,27]. Additionally, at 1052 and 1240 cm^−1^, band bending is related to the hydroxyl groups coordinated with the oxide structure [25,26].

**Figure 2:**
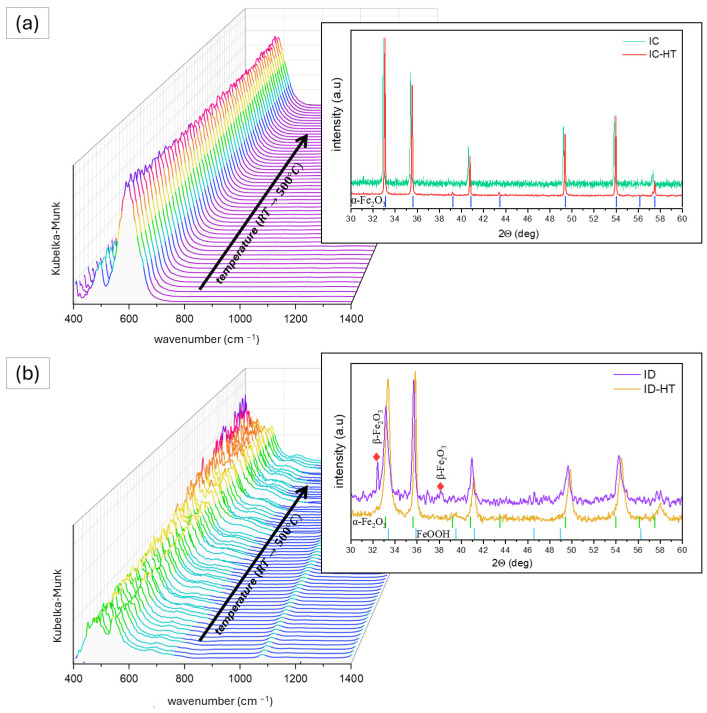
FTIR-drifts spectra with XRD analysis (inset) of the iron oxide-based materials in the form of (**a**) cubes and (**b**) discs.

The same phase composition can be observed for the cubes. The diffuse reflectance infrared Fourier transform spectroscopy (DRIFTS) temperature studies, as well as the XRD analysis, are shown in Figure 2a. The annealing process leads to increased crystallinity, which affects band sharpening. The XRD phase composition before and after thermal treatment shows that the recorded pattern can be only assigned to α-Fe_2_O_3_ (PDF# 01-089-0598). The slight shift of the reflection toward the reference suggests the zinc ions are incorporated into the structure.

On the other hand, bands characteristic of Fe-O interactions are visible; nevertheless, due to the orientation of some planes, the ratio of the bands at 476 and 571 cm^−1^ is reversed. Moreover, the addition of ions during synthesis (Al^3+^) results in a band shift toward the lower wavenumbers [27,28]. This is except for the typical vibrations mode for the hematite disc-like samples after synthesis (Figure 2b), a strong mode associated with OH bending (670 cm^−1^), which can be attributed to the presence of the FeOOH phase [29], as well as the bands at 1063 and 1096 cm^−1^. However, the last two diminish with increasing temperatures. Changes in the phase composition were also confirmed by the XRD measurements. In addition to hematite, orthorhombic lepidocrocite (γ-FeOOH, PDF# 01-074-1877) and cubic β-Fe_2_O_3_ (PDF# 01-076-1821) are present. The annealing at 500 °C in the air affects their transformation into pure α-Fe_2_O_3_.

### 2.2. Photocatalytic Tests

To demonstrate the viability of the cube and disc-like Fe_2_O_3_ nanocrystals toward cationic and anionic dyes, adsorption studies and photoactivity tests involving the analyzed shaped iron oxides were conducted using two types of dye: cationic methylene blue (MB) and anionic methyl orange (MO).

**Figure 3:**
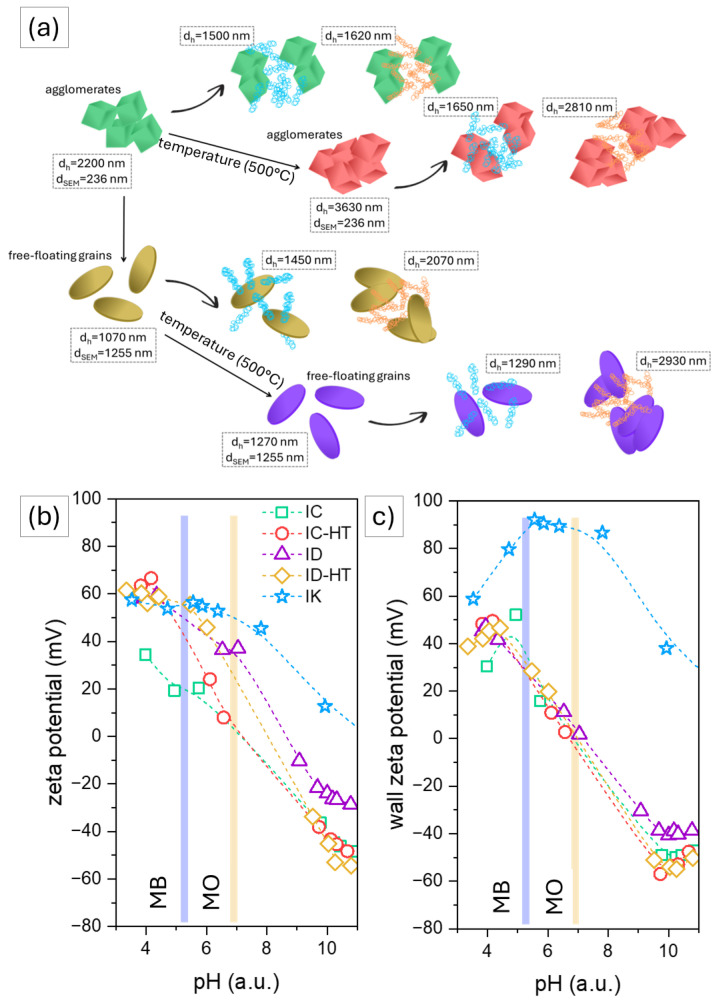
(**a**) Dispersion scheme of Fe_2_O_3_ nanomaterials (powder concentration: 0.25 gL^−1^) in water and organic dye (MB, MO) solutions (dye concentration: 3 × 10^−5^ molL^−1^), and (**b**,**c**) zeta and wall zeta potential of the analyzed samples, respectively (d_SEM_—particle diameter from SEM measurements). The blue and orange chains denote MB and MO molecules, respectively.

The explanation for the differential behavior of the materials depending on the shape adopted, as well as on how the materials were obtained, can be found in the number and type of available active sites on the surface. When nanoparticles are dispersed in water (Figure 3a), their surface is automatically covered with hydroxyl groups. The determined hydrodynamic diameter (d_h_) allows for evaluating the nanoparticles’ tendency to agglomerate and, indirectly, the probability that a given dye is adsorbed on the surface of the adsorbent. Surprisingly, the FC powders are characterized by higher d_h_ than discs. However, considering their smaller size, the probability of their agglomeration is higher.

Based on the differences between the d_h_ values in the water and dye solutions, it can be concluded that for cubes, in contrast to the water, the presence of organic dye affects the breakdown of the agglomerates into smaller groups. The higher the level of dye adsorption on the material’s surface, the smaller the diameter observed for a given group should be. For the powders after synthesis (IC), the analyzed differences are similar, but annealing at 500 °C (IC-HT) results in better adsorption of methylene blue than in MO.

Starkly different data were obtained for the disc-like iron oxides. Regardless of the type of the dye, the observed d_h_ is similar or larger in the dye solution than in water. In the first case, this may be due to the free adsorption of dye molecules on the surface of the grains, while in the second case, agglomeration of the photocatalyst and simultaneous adsorption of the dye may occur. Despite the thermal treatment, the observed powder behavior is different than that of cubes. This is particularly noticeable in the case of MO adsorption. Two points should be considered: (1) surface dehydroxylation related to the phase transformation from FeOOH to hematite and (2) modification of the defective centers located in the materials’ crystal facets (especially at the (2–10) facet). According to Amami et al. [30], oxygen vacancies are dominant defects in the case of hematite and are involved in the oxygen self-diffusion mechanism. The phase transformation of lepidocrocite (γ-FeOOH) and the recrystallization of α-Fe_2_O_3_ (Figure 2b) can provide oxygen vacancies and interstitial iron appearance. However, more information may be gathered by comparing the DLS results with data collected from the surface state.

The measurements of zeta and wall zeta potentials are highly sensitive techniques that allow the observation of how the surface charge changes with the pH of the solution. Figure 3b,c shows the zeta and wall zeta characteristics of the analyzed iron oxide samples, respectively. In the first case (Figure 3b), despite the materials’ shape, their surface is protonated (FeOH^2+^) for a wide range of the solution’s pH (below 9 for IC, and below 11 for ID and IK) and, more importantly, the value of the zeta potential is higher than 30 mV. Therefore, the samples can be assumed to be stable. This confirms the effect of the adsorption of individual dyes on the shape obtained and the heat treatment process. After annealing, at 500 °C a shift of the isoelectric point (point of zero charge) toward lower values can be observed. The highest zeta potentials are observed for disc-shaped iron oxides, even after thermal treatment. When compared to the obtained results for the above-mentioned material and commercial powder (IK), it is clear that the presence of different energies promotes better homogeneity of the surface charge.

Another piece of information that is complementary to the observed behavior of the materials is the wall zeta. These measurements are sensitive toward the adsorption of various soluble components. It can be used to track both the kinetics of adsorption and to construct an adsorption isotherm [31]. The potential value depends on the properties of the electrolyte (pH, ionic strength, additive concentration) and the tested material (shape, porosity, electrical conductivity, and surface condition), as well as the measurement time and temperature. Figure 3c presents the wall zeta potential as a function of pH. It is clear that, for cube-like materials, the annealing process does not affect their state and ability to adsorb molecules. On the other hand, the thermal treatment did have an impact on the surface of the discs. The values obtained in the alkaline solution are much lower than those obtained without annealing. According to the data obtained, it can be assumed that the adsorption process for this powder before and after annealing is determined by different factors related to the surface state.

#### 2.2.1. Cationic Dye

**Figure 4:**
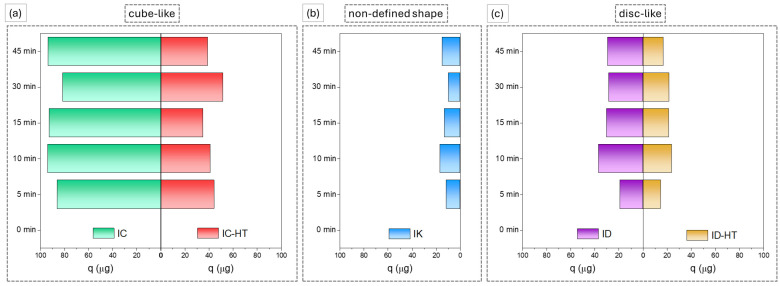
Adsorption kinetics of methylene blue on Fe_2_O_3_ adsorbents (powder concentration: 1.25 gL^−1^) as well as the amount of the adsorbed dye on the surface of samples after a particular processing time: (**a**) cube-like materials, (**b**) the powder with non-defined shape, (**c**) disc-like samples.

The kinetic changes of methylene blue adsorption after various time intervals are shown in Figure 4.

As predicted by the dls measurements, the cube-like powders exhibit the highest affinity for methylene blue, both in comparison to the IK reference sample and the disc-like materials. After annealing powders, the adsorption of the MB is changed. For the IC and IC-HT samples composed of pure hematite, the decrease in the adsorption capacity for IC-HT may be attributed to the loss of the hydroxyl radicals, which may act as additional active sites (bridges) between the dye and photocatalyst. Nevertheless, this value is still higher than that for disc-shaped materials. Despite the relative bulk-to-surface concentration ratio, and exposition of the (110) plane, which is considered as the adsorption reaction preferred plane [32], the performance of discs is not as good as assumed.

Adsorption is the initial stage of the photodegradation reaction. However, it is not the only parameter responsible for the efficiency of dye decomposition. Therefore, after 30 min of mixing in the dark, tests involving the photodecomposition of dyes were performed.

**Figure 5:**
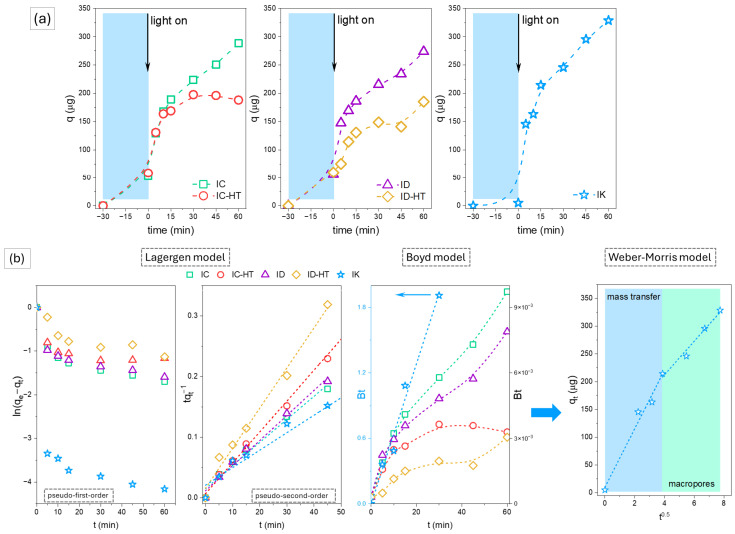
Photocatalytic activity of the materials obtained, determined by (**a**) the amount of photodecomposed dye (µg) and (**b**) analysis of the mechanism determining the catalytic process using the Lagergen, Boyd, and Weber-Morris model (powder concentration: 1.25 gL^−1^, dye concentration: 3 × 10^−5^ molL^−1^).

Figure 5a shows the amount of photocomposed methylene blue with an increasing time of illumination in the presence of photocatalysts. It can be seen that all the Fe_2_O_3_ materials—cubes (IC, IC-HT), discs (ID, ID-HT), and commercial powder (IK)—demonstrate a good degradation rate. It is a fact that regardless of how much dye was adsorbed on the surface, the observed rate of change of its concentration in solution is comparable for as-synthesized and IK samples. The difference is for the heat-treated samples, which may suggest a different mechanism. Therefore, to determine the cause of the changes, Lagergen kinetics models were used [33,34].

The pseudo-first-order defines that the mechanism is based on diffusion through the following boundary [33,34]:

(1) $$\ln\left(q_{e}-q_{t}\right)={\mathrm{lnq}}_{e}-k_{1}t$$

where q_e_ is the amount of the adsorbed at the time 0, q_t_ is the amount photodegraded at the time t, and k_1_ is defined as a rate constant. The plot ln(q_e_ − q_t_) vs. t should give a straight line with slope k_1_ and intercept lnq_e_. However, the observed curves (Figure 5b) suggest that the model adopted is incorrect.

The pseudo-second-order, described by Lagergen, assumes that chemisorption is a rate-controlling step [33,35]:

(2) $$\frac{t}{q_{t}}=\frac{1}{k_{2}q_{e}^{2}}+\frac{1}{q_{e}}t$$

where k_2_ (the pseudo-second-rate constant) can be determined from the slope of the plot of t/q_t_ vs. t, and q_e_ can be determined from the intercept.

For all samples, the linear relation was obtained. It can be claimed that the particle surface plays a key role in the photodegradation process. The calculated values of k_2_ and q_e_ are collected in Table 1.

The rate of the photodegradation process depends on the amount of adsorbed dye and, thus, the available active sites. IC and IK show a good ratio of the amount of the initially adsorbed dye to the decomposition rate when the material is illuminated. As a result of the process of materials annealing, surface changes (removal of pre-adsorbed hydroxyl radicals) occur and thus the process parameters deteriorate. The opposite is shown for disc-shaped particles. For ID-HT, the determined rate of change is the highest, but at the same time, this is due to the small amount of initially adsorbed dye. This may be the effect of surface dehydration and phase transformation.

The role of the particle structure like the rate control step can be determined by Boyd and Webber-Moris kinetic models (Figure 5b) [33].

For q_t_/q_e_ less than 0.85, the Boyd plot, also assigned in such conditions as the Reichberg equation, can be written in the following form [36]:

(3) $$B_{t}=\pi {\left(1-\sqrt{1-\frac{\pi }{3}\frac{q_{t}}{q_{e}}}\right)}^{2}$$

where B_t_ is a time constant. For the straight line fitted to the linear range of B_t_ vs. t, the process is governed by the intra-particle diffusion model (sample porosity), or by film diffusion otherwise. As-synthesized iron oxides show mixed mechanisms, which can be related to the presence of a pre-adsorbed hydroxyl radical layer or different phase composition. On the other hand, annealing unravels the original background of their photocatalytic activity, which is strictly affiliated with the chemisorption of the organic dye. A completely different behavior is observed for commercial powder, which may be due to a wide grain size distribution.

The Webber-Morris plot allows for identifying the steps involved in adsorption, and therefore photodegradation process. The model can be expressed as follows [37,38]:

(4) $$q_{t}=k_{\mathrm{id}}t^{0.5}+C$$

For IK, the plot q_t_ vs. t^0.5^ is given by two regions, which may be assigned to the external mass transfer followed by macropore diffusion. The results obtained and the SEM observations remain in agreement. The commercial powder shows a dual nature, which is due to the presence of two different groups of grains.

#### 2.2.2. Anionic Dye

**Figure 6:**
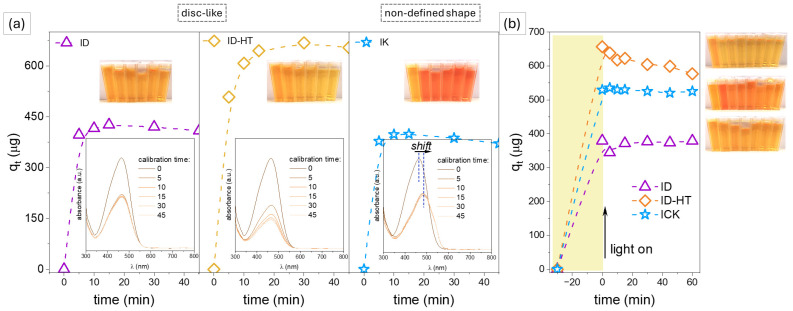
**(a**) Adsorption kinetics of methyl orange on Fe_2_O_3_ adsorbents (powder concentration: 1.25 gL^−1^) as well as (**b**) the amount of the adsorbed dye on the surface of samples after a particular processing time.

**Figure 7:**
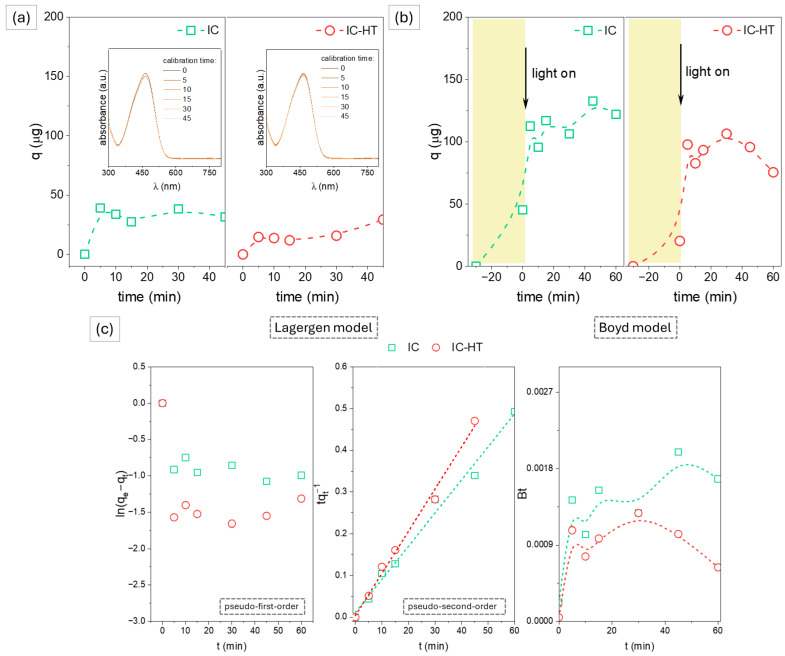
(**a**) Adsorption capacity of the cube-like hematite particles (the insets show the changes in the absorbance of the MO solutions), (**b**) photocatalytic activity determined by the amount of photodecomposed dye (µg), and (**c**) analysis of the mechanism determining the catalytic process using the Lagergen and Boyd model (powder concentration: 1.25 gL^−1^, dye concentration: 3 × 10^−5^ molL^−1^).

The adsorption–desorption test in the presence of methyl orange is shown in Figure 6a. The highest affinity toward methyl orange was observed for the disc-like powders (ID-HT). After annealing, the adsorption properties of the disc change significantly, which is in good correlation with the results of wall zeta measurements. This behavior should be closely associated with the phase transition. IC and IC-HT (Figure 7a) samples are composed of pure hematite and exhibit a much lower adsorption capacity than ID and ID-HT. The performance of disc-shaped powders may be improved by tuning the relative bulk-to-surface ratio. Disc-like materials are characterized by a large surface area, which is closely related to their shape. A significant disproportion between disc dimensions (1255 nm vs. 141 nm) and large exposition of the (110) plane facilitate the adsorption reactions [32]. Surprisingly, no change in dye concentration was observed after illumination of the solution (Figure 6b). For both disc-shaped powders and IK, the amount of MO absorbed on the surface after 60 min of illumination is constant, which means that no redox reactions take place, even in the presence of hydrogen peroxide, whose purpose is to promote Fenton reactions.

On the other hand, despite the low adsorption capacity of the MO (Figure 7a), IC and IC-HT are photoactive (Figure 7b). The observed efficiency of the photodegradation process is lower than that of MB. Nevertheless, a comparative analysis of the catalytic models for both dyes confirmed the same mechanism (Figure 7c).

The key element is the chemisorption of the dye on the surface of the oxides. This is in contradiction to the other systems. However, this does not explain the photoactivity of the materials compared to the other forms studied.

For this purpose, additional studies related to the determination of the energy gap of the materials and the position of the valence and conductivity bands were performed.

**Figure 8:**
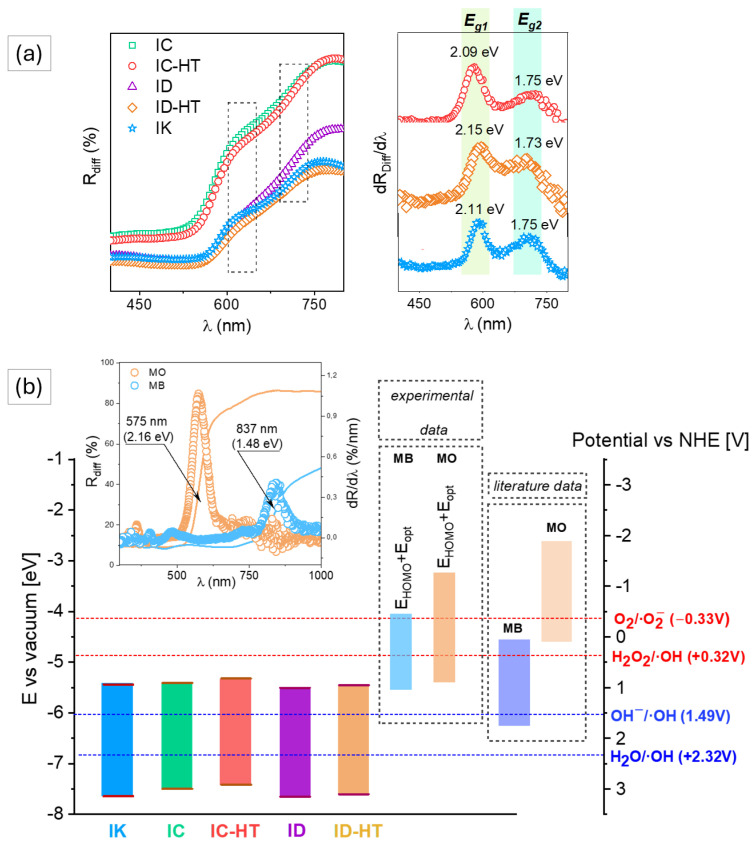
(**a**) Different optical reflectance R_diff_ (λ) spectra recorded for Fe_2_O_3_ materials with the corresponding differential spectra dR_dif_/dλ, and (**b**) energy level diagram of Fe_2_O_3_ concerning the potential of reactive oxygen species (ROS) and the HOMO-LUMO levels of dyes. The inset presents differential spectra dR_dif_/dλ for organic dye powders (MO, MB).

The optical spectra of the analyzed iron oxides are shown in Figure 8a. The annealing temperature does not affect the position of the absorption edge that occurs in the range between 550 and 730 nm. For nanocrystals in the form of cubes (IC and IC-HT), the fundamental adsorption edge shifts toward shorter wavelengths. The reflectance, R_diff_, as a function of wavelength was analyzed by applying differential reflectance spectra. Figure 8a presents some results obtained using the band gap determination method. The value of the bandgap energy, E_g_, corresponds to the point at which the maximum of dR_diff_/dλ occurs [39]. Regardless of the form of the material, there are two values of E_g_. The higher (2.09–2.15 eV) value originates from a direct transition in α-Fe_2_O_3_, whereas the lower one in the range of 1.73–1.75 eV can be attributed to the indirect transition.

Apart from the interaction between the dye molecules and photocatalysts (adsorption) and an appropriate band gap, the position of the conduction band (CB) and (VB) edges also plays a key role in the application of semiconductors in the field of photocatalysis. In this work, the positions of the CB and VB edges were calculated using the approach proposed by Butler and Ginley [40] and modified by Zhang and Jaroniec [41]:

(5) $$E_{\mathrm{CB}}=-\chi +\frac{1}{2}E_{g}-2.303\frac{\mathrm{RT}}{F}{\mathrm{pH}}_{\mathrm{PZC}}$$

(6) $$E_{\mathrm{VB}}=E_{\mathrm{CB}}-E_{g}$$

where χ is the Sanderson electronegativity of the semiconductor (χ = 5.88 for Fe_2_O_3_), R stands for the universal gas constant, T is the absolute temperature, F is the Faraday constant, and pH_PZC_ is the point of zero charge. The value of the isoelectric point was determined based on the dependence of the zeta potential on pH for iron oxides (Figure 3b).

**Figure 9:**
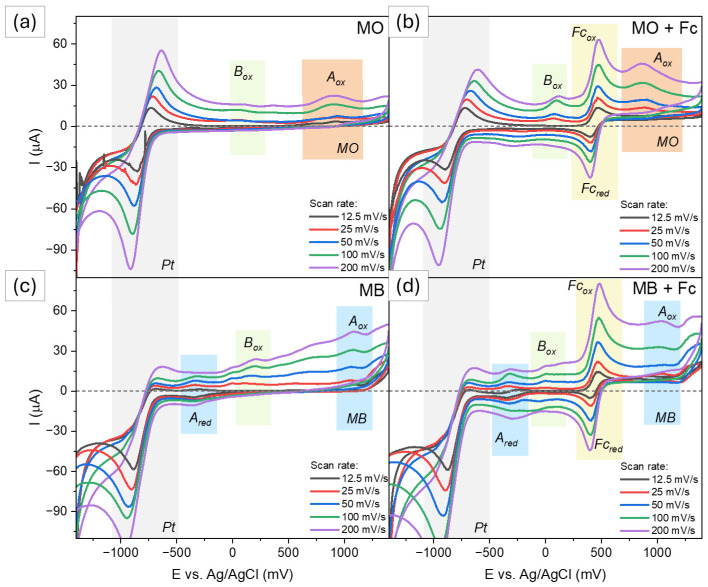
Cyclic voltammograms were recorded for (**a**) methyl orange (1 mM), (**b**) methyl orange (1 mM) with dissolved ferrocene (3 mg/15 mL) as the internal standard, (**c**) methylene blue (1 mM), and (**d**) methylene blue solution (1 mM) containing ferrocene (3 mg/15 mL). As the electrolyte, the 0.1 M solution of TBAPF_6_ in acetonitrile was used.

The HOMO energies of dyes were determined based on CV measurements with ferrocene (Fc) as an internal standard. Figure 9 presents the recorded cyclic voltammograms for the investigated dyes’ systems with/without ferrocene. The oxidation (ox) and reduction (red) peaks related to the reactions of MO (ca. 0.9 V vs. Ag/AgCl) and MB (ca. 1.05 V and −0.35 V vs. Ag/AgCl) dyes are labeled A_ox_ and A_red_, while those corresponding to the redox processes of ferrocene (ca. 0.4–0.5 V vs. Ag/AgCl) are marked as Fc_ox_ and Fc_red_. The peaks originating from the electrochemical reactions of the Pt electrode (ca. from −0.9 to −0.6 V vs. Ag/AgCl) [42] and the electrolyte (B_ox_), which are of no relevance to the HOMO determination procedure, are marked with green and grey colors, respectively. Based on the conducted measurements, the A_ox_ peaks related to the HOMO levels were recorded for both dyes, while the reduction peak connected with the LUMO level could only be observed for the MB (the A_red_ potential of MO most probably exceeds the accessible potential window of the applied electrolyte). Therefore, the LUMO energies (E_(LUMO)_) were estimated based on the obtained HOMO-LUMO energy gaps (E_opt_) from the diffuse reflectance spectra of the dye powders (Figure 8b) by adding the E_opt_ value to the determined HOMO energy levels (E_(HOMO)_). 

The procedure for E_(HOMO)_ determination was the following. Firstly, the potential scale of CVs presented in Figure 9a,c was normalized relative to the ferrocene redox potential (E_redox_,_Fc_), which was established according to the following formula:

(7) $$E_{\mathrm{redox},\;\mathrm{Fc}}=\frac{E_{{\mathrm{Fc}}_{\left(\mathrm{ox}\right)}}+E_{{\mathrm{Fc}}_{\left(\mathrm{red}\right)}}}{2}$$

where E_Fc(ox)_ and E_Fc(red)_ denote the peak potential for Fc_ox_ and Fc_red_, respectively. The shift scale was corrected by the ΔE value, which results from the shifts of A_ox_ peaks in the systems containing ferrocene. The ΔE value was determined by subtracting the A_ox_ peak potential (E_Aox_) in Figure 9a,c from that presented in Figure 9b,d for each dye. Consequently, the potential scale was shifted according to the following equation:

(8) $$E\left(\mathrm{vs}.\;\mathrm{Fc}\right)=E\left(\mathrm{vs}.\mathrm{Ag}/\mathrm{AgCl}\right)-E_{\mathrm{redox},\;\mathrm{Fc}}\left(\mathrm{vs}.\;\mathrm{Ag}/\mathrm{AgCl}\right)+\Delta E$$

Subsequently, for CVs with normalized potential scales, the redox potential values (E_redox_) for A_ox_ peaks were approximated by applying the following formula:

(9) $$E_{\mathrm{redox}}=\frac{E_{p/2}+E_{p}}{2}$$

where E_p_ denotes the peak potential, and E_p/2_ represents the potential value corresponding to the determined half-peak current. The obtained E_redox_ values were converted to the vacuum scale by using the following equation:

(10) $$E\left(\mathrm{vs}.\;\mathrm{vacuum}\;\left[\mathrm{eV}\right]\right)=-\left({\mathrm{eE}}_{\mathrm{redox}}\left(\mathrm{vs}.\;\mathrm{Fc}\;\left[V\right]\right)-\left(-4.99\;\left[\mathrm{eV}\right]\right)\right)$$

in which e is the elementary charge, and the value of −4.99 eV represents the redox potential of ferrocene vs. vacuum. For both dyes, the calculations were performed for each scan rate. The results are collated in Table 2.

Figure 8b presents the position of the valence and conduction band levels of Fe_2_O_3_ photocatalysts and the potentials of reactive oxygen species (ROS) along with the LUMO and HOMO levels of MB and MO. A semiconductor for environmental applications should exhibit high photoactivity with regard to both the reduction of O_2_ and the oxidation of surface H_2_O/hydroxyl group, which allows it to efficiently generate ROS. However, this criterion is usually met by wide-band-gap semiconductors with suitable energy band structures, such as TiO_2_ or SrTiO_3_. The positions of conduction (CB) and valence (VB) band edges for Fe_2_O_3_, which is a visible-light-driven semiconductor, determine its capacity for the oxidation of H_2_O/OH groups to ^·^OH radicals. The energy levels of the lowest unoccupied molecular orbital (LUMO) of dye show that their photogenerated electrons are capable of reducing O_2_ (in the case of methyl orange (MO)) and H_2_O_2_ (for methylene blue (MB)). However, the positions of the highest occupied molecular orbital (HOMO) levels of MB and MO indicate that photogenerated holes cannot drive the oxidation of H_2_O to ·OH radicals. Only for methylene blue can photogenerated holes participate in the oxidation of OH- groups. The above data confirm that despite the slight adsorption of methyl blue compared to MO, the decomposition process under illumination in the UV-ViS range does take place.

With reference to the data, the process of photocatalysis should not take place for any of the tested materials in the presence of methyl orange. However, the experimentally determined position of the HOMO and LUMO bands for MO made it possible to observe an overcrossing of the CB levels from IC and IC-HT relative to the HOMO of OM.

Another important issue is the dye self-sensitization process [43]. Both MO and MB, upon visible light irradiation, can absorb radiation of a particular energy, and thus photoexcitement of electrons can occur. This charge may be injected from the dye LUMO level to the CB of the photocatalysts. However, herein, a key factor in the degradation of dyes is Fenton reactions (H_2_O_2_ used), responsible for generating hydroxyl radicals involved in redox reactions [23]. Without this compound, no change in dye concentration is observed after illumination with light in the visible light range.

Crystal facet engineering affects the tailored superficial properties that can unlock the activity in photodegradation. In the case of the hematite, studies show that the adsorption reaction can be considered mostly on particular facets like (100), (110), (012), or (104), where the single coordinated hydroxyl radicals groups may be preset [32]. This would remain consistent with the results obtained experimentally, especially with the large surface area of the disc-shaped particle (plane (110)). On the other hand, the crystal orientation may affect the energy band structure (CB and VB levels) [44]. As a result of this phenomenon, it is possible to observe small changes in the concentration of the dye during illumination. The arrangement of ID, ID-HT, and IK bands eliminates these materials as photocatalysts for MO; nevertheless, they can be used as adsorbents of this compound.

**Figure 10:**
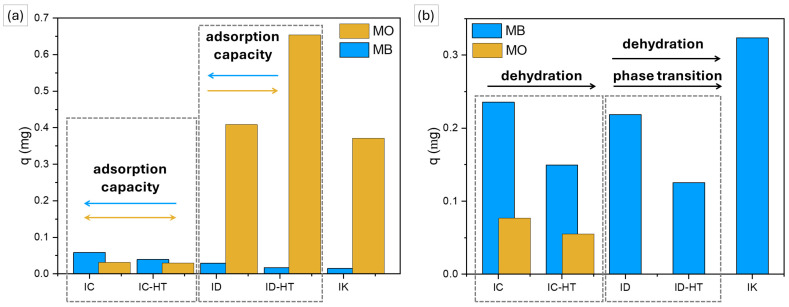
Amount of the (**a**) adsorbed dyes after 45 min, and (**b**) the photodecomposed MB and MO after 60 min under visible light illumination.

Summarizing all performed research and obtained results, it can be concluded that cube-shaped particles serve as better adsorbers for cationic dyes, e.g., MB. This is especially true for IC due to the presence of physically sorbed water on its surface (Figure 10a). Disc-like iron oxides with a stable hematite structure (ID-HT) can be applied to remove contaminants in the form of OM using adsorption processes. Despite the structure and type of the dye, as-synthesized materials exhibit better catalytic properties (Figure 10b). This is influenced by the surface state of the obtained materials along with the heterostructural nature of the system, facilitating the transfer of photoexcited electron carriers (in the case of discs).

## 3. Experiment

### 3.1. Synthesis

**Scheme 1:**
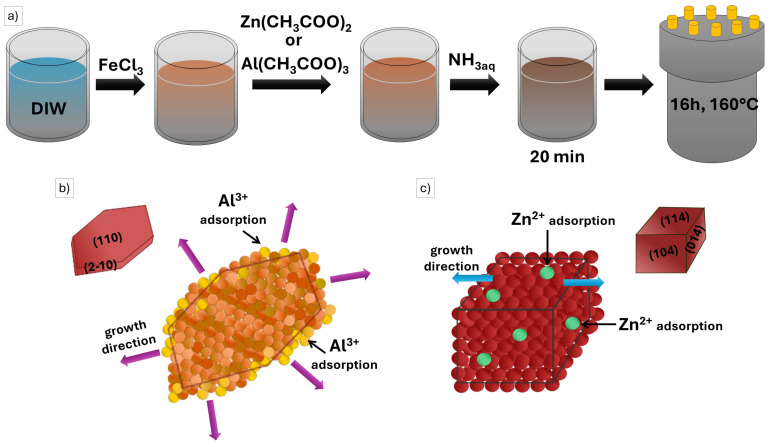
(**a**) Schematic illustration of the synthesis mechanism, with a graphical explanation of the ions’ affection to iron oxide grains growth in the shape of (**b**) disc and (**c**) cubes.

Nanomaterials were prepared via a metal ion-mediated hydrothermal route. An acetate precursor (zinc acetate dihydrate, POCH, Gliwice, Poland, and aluminum acetate, Sigma-Aldrich (Merck), Darmstadt, Germany) was added to the 80 mL solution containing the iron (III) chloride nonahydrate (POCH, Gliwice, Poland) to obtain cubes and disc, respectively. Afterward, the ammonium solution (POCH, Gliwice, Poland) was added. Before the mixture was placed in the Teflon-line stainless-steel autoclave for 16 h at 160 °C, it was stirred for 20 min. The obtained powders were centrifuged, rinsed several times with ethanol (POCH, Poland) and distilled water solution (50/50 *v*/*v*), and dried at 60 °C for 12 h. During nucleation and crystallite growth, Al^3+^ ions adsorb on the (2–10) planes, forcing growth on grains along the plane (110). When Zn^2+^ ions are used as a modifier, they undergo adsorption on the planes (104), which affects their elongation. As a result of the reaction between the Al^3+^ and Zn^2+^ with hydroxyl ions and ammonia, respectively, complexes are formed and leached out. The schematic illustration of the synthesis parameters and the affection of ions is presented in Scheme 1.

After the synthesis, some of the obtained samples were annealed for 3 h in the air at 500 °C. Additionally, an iron (III) oxide commercial powder was used as a reference material. All detailed information devoted to the synthesis and sample designations is summarized in Table 3.

### 3.2. Materials Characterization

The morphology of the samples was performed by means of scanning electron microscopy (SEM, ThermoFisher Scientific Scios 2, Waltham, MA, USA). The grain size of the powders was determined using the ImageJ program (version 1.54g).

The crystal structure of the obtained materials was investigated using an X’Pert MPD diffractometer (Malvern Panalytical Ltd., Malvern, UK). The system worked in Bragg–Brentanno geometry. Phase identification was carried out using X’Pert HighScore Plus software (version 3.0.4) and the Powder Diffraction File (PDF-2).

The structural properties of the samples were also examined by performing Fourier transform infrared spectroscopy (FTIR) temperature studies using the diffuse reflection technique with a Bruker Vertex 70v spectrometer (Bruker, Berlin, Germany) equipped with a Harrick “Praying Mantis” DRIFTS (diffuse reflectance infrared Fourier transform spectroscopy) attachment combined with a high-temperature reaction chamber (Harrick Scientific Products Inc., Orpington, UK). Measurements were carried out in the mid-infrared range (4000–400 cm^−1^). Kubelka–Munk spectra were recorded over 64 scans with a resolution of 4 cm^−1^.

Nitrogen adsorption/desorption analysis was carried out at –196 °C using an Autosorb-1 Quantachrome surface area/pore size analyzer (Micrometrics, Norcross, GA, USA). Before sorption measurements, the samples were preheated and degassed in a vacuum at 100 °C for 18 h.

The optical properties of the prepared materials were analyzed using a double-beam Jasco UV-ViS-NIR V-670 spectrophotometer (Jasco, Oklahoma City, OK, USA) with a 150 mm integrating sphere. The band gap values were determined from the first derivative of diffuse reflectance spectra and calculated as in our previous work [23].

The dynamic light scattering (DLS) technique was used to measure the hydrodynamic diameter (d_h_) of particle dispersions in aqueous and organic dye solutions. Methylene blue (MB) and methyl orange (MO) were used as representatives of cationic and anionic dyes, respectively. The electrophoretic light scattering (ELS) technique was used to characterize zeta and wall zeta potentials. The values were determined using Zetasizer Pro (Malvern Panalytical Ltd., Malvern, UK).

### 3.3. Photocatalytic Activity

The dye adsorption properties of the prepared samples were investigated based on the adsorption–desorption kinetics of MB and MO. For each measurement, Fe_2_O_3_ adsorbent (0.125 g of IC, ID, IC-HT, ID-HT, or IK) was dispersed in the dye solution (3 × 10^−5^ mol L^−1^, 100 mL). The suspension was continuously stirred for 45 min in the dark and at room temperature. At given time intervals, the suspension was collected (4 mL), filtered, and analyzed using the Jasco UV-ViS-NIR V-670 spectrophotometer (Jasco, Oklahoma City, OK, USA). Measurements of absorbance spectra were performed in the wavelength range from 250 to 800 nm. The concentrations of MB and MO were determined by checking the maximum absorbance value at 664 and 464 nm, respectively.

The amount of the adsorbed dye from the solution (q) was calculated according to the following equation:

(11) $$q=\left(C_{0}-C_{t}\right){\mathrm{VM}}_{d}$$

where C_0_ is the initial dye concentration, C_t_ is the concentration of dye at time t (min), V is the volume of the solution, and M_d_ is the molar weight of the dye.

The photocatalytic experiments were carried out in a self-made cylindrical photoreactor comprising twelve Philips TL 8W/54-765 lamps (Amsterdam, Netherlands) and a quartz reaction vessel (power of the light source 52.4 mWcm^−2^). For each experiment, the photocatalyst (IC, ID, IC-HT, ID-HT, or IK) concentration in the dye (3∙10^−5^ mol L^−1^ MB or MO) solution was the same as for adsorption kinetics measurements (1.25 g L^−1^). Before illumination with visible light, the mixture was magnetically stirred for 30 min, and then H_2_O_2_ (30%, 3 mL) was added. During 1 h illumination, the suspension was collected at given time intervals and analyzed in the same manner as during the dye adsorption measurements.

The lowest unoccupied molecular orbital (LUMO) and highest occupied orbital (HOMO) energy levels (E_(LUMO)_, E_(HOMO)_) of MB and MO were investigated by conducting cyclic voltammetry (CV) measurements with ferrocene (Fc) as an internal standard. The measurements were carried out with an M161 electrochemical analyzer (MTM-ANKO, Krakow, Poland) with a conventional three-electrode cell, consisting of a glassy carbon working electrode (GCE), a platinum wire (Pt) as a counter electrode, and a silver chloride electrode (Ag/AgCl, 3 M KCl) as the reference one. As a supporting electrolyte, the solution of acetonitrile containing tetrabutylammonium hexafluorophosphate (TBAPF_6_, 0.1 M) was used. At the end of the tests with dyes (1 mM), 3 mL of ferrocene was added to the system (15 mL), and cyclic voltammograms were re-measured. Analyses were carried out by applying the potential range between −1400 and 1400 mV with the scan rate from 12.5 to 200 mVs^−1^. The reference potential (E vs. Ag/AgCl) was normalized according to the ferrocene redox potential (E_redox,Fc_) by shifting the E scale to set E_redox,Fc_ as 0 V (E vs. Fc), applying a correction from the shift of dyes signals in the presence of ferrocene (ΔE) [42]. The determined energy levels from CV measurements were converted to the vacuum scale by assuming E_redox, Fc_ vs. vacuum is −4.99 eV. The recorded signals enabled us to determine dyes’ HOMO levels. Therefore, the HOMO-LUMO energy gap (E_opt_) of MB and OM was calculated from the UV-ViS spectroscopy analysis by means of the first-derivative method. The determined E_opt_ values were added to the E_(HOMO)_ energy levels to approximate E_(LUMO)_.

## 4. Conclusions

The synthesis parameters (ion additives, annealing) affect the shape, phase composition, and surface state (presence of hydroxyl groups) of the obtained materials. Applying Zn^2+^ and Al^3+^ ions during synthesis yields cube and disc-like samples, respectively. Annealing the samples changes their surface state and, in the case of discs, the phase composition. However, thermal treatment does not affect the optical properties of either type of material.

The dispersions of the analyzed powders are stable over a wide pH range (ZP > 30 mV). Differences in the obtained zeta potential-pH curves are mainly due to the shape of the samples. Nevertheless, annealing also contributes to the shift in isoelectric point values. Wall zeta potential (WZP) measurements showed that annealing the discs changes their surface state and ability to adsorb molecules. This was confirmed via hydrodynamic diameter (d_h_) measurements and adsorption–desorption tests.

For disc-like samples, regardless of whether they are annealed or not, the hydrodynamic diameter is higher in the dye solutions than in water. This increase is related to the adsorption of dyes onto the surface of the discs. Starkly different results were obtained for the cubes, which have smaller d_h_ values in the dye solutions. This decrease is likely related to the breakdown of agglomerates into smaller groups in the presence of the dyes. As predicted from the zeta potential measurements, the affinity of the powders toward anionic methyl orange (MO) is higher than that of cationic methylene blue (MB), which is a consequence of the surface state of the samples.

The heterogeneous photocatalytic reaction is inherently related to dye adsorption on the surface of the semiconductor as the first step of a photodecomposition process. In addition, the properties of the photocatalyst, such as the surface structure, optical band gap, as well as the proper position of the band edges concerning the redox potential levels, strongly affect photodegradation activity. IC and IC-HT powders, despite their low adsorption capacity due to the presence of their energy-equivalent walls, can be used as photocatalysts for cationic and anionic dyes. The crystal orientation of the cube-shaped particles affects the energy band structure (CB and VB levels). Therefore, it is possible to observe small changes in the concentration of the MO during illumination.

## Figures and Tables

**Table 1:** Lageregen kinetics model parameters.

Parameter	IC	IC-HT	ID	ID-HT	IK
**k_2_ (gmin^−1^)**	0.17	0.29	0.21	0.10	0.17
**q_e_ (g)**	0.0026	0.0020	0.0024	0.0015	0.0035

**Table 2:** Summary of the determined redox potentials for MO and MB, together with the calculated HOMO and LUMO energy levels.

Scan Rate (mVs^−1^)	MO				MB			
	E_redox_ (V)	E_(HOMO)_ (V)	E_opt_ (eV)	E_(LUMO)_^1^ (V)	E_redox_ (V)	E_(HOMO)_ (V)	E_opt_ (eV)	E_(LUMO)_^1^ (V)
**12.5**	n/a	n/a	2.16	−3.24	n/a	n/a	1.48	−4.07
**25**	0.44	−5.43			0.57	−5.56		
**50**	0.44	−5.43			0.56	−5.55		
**100**	0.38	−5.37			0.55	−5.54		
**200**	0.37	−5.36			0.56	−5.55		
**average**	0.41	−5.40			0.56	−5.55		

^1^ E_(LUMO)_ were obtained by adding E_opt_ to the average E_(HOMO)_ value.

**Table 3:** Synthesis parameters and sample designations.

Synthesis Parameters	Cube-like		Disc-like		Reference
	IC	IC-HT	ID	ID-HT	IK
**metal precursor**	16 mmol FeCl_3_·9H_2_O				commercial powder Chempur, >96%, Fe_2_O_3_
**electrolyte**	80 mL distilled water				
**modifier**	8 mmol (CH_3_COO)_2_Zn·2H_2_O		8 mmol (CH_3_COO)_3_Al		
**precipitating agent**	80 mL NH_3aq_, 25%				
**reaction**	160 °C, 16 h				
**post-treatment**	-	500 °C, 180 min, air	-	500 °C, 180 min, air	-

## Data Availability

Dataset available upon request from the authors.

## References

[1] Tong H., Ouyang S., Bi Y., Umezawa N., Oshikiri M., Ye J. (2012). Nano-Photocatalytic Materials: Possibilities and Challenges. Adv. Mater..

[2] Fresno F., Portela R., Suárez S., Coronado J.M. (2014). Photocatalytic Materials: Recent Achievements and near Future Trends. J. Mater. Chem. A Mater..

[3] Mills A., Le Hunte S. (1997). An Overview of Semiconductor Photocatalysis. J. Photochem. Photobiol. A Chem..

[4] Gusain R., Gupta K., Joshi P., Khatri O.P. (2019). Adsorptive Removal and Photocatalytic Degradation of Organic Pollutants Using Metal Oxides and Their Composites: A Comprehensive Review. Adv. Colloid. Interface Sci..

[5] Hernández-Alonso M.D., Fresno F., Suárez S., Coronado J.M. (2009). Development of Alternative Photocatalysts to TiO2: Challenges and Opportunities. Energy Environ. Sci..

[6] Li J., Wu N. (2015). Semiconductor-Based Photocatalysts and Photoelectrochemical Cells for Solar Fuel Generation: A Review. Catal. Sci. Technol..

[7] Jing L., Zhou W., Tian G., Fu H. (2013). Surface Tuning for Oxide-Based Nanomaterials as Efficient Photocatalysts. Chem. Soc. Rev..

[8] Li X., Yu J., Jaroniec M. (2016). Hierarchical Photocatalysts. Chem. Soc. Rev..

[9] Daghrir R., Drogui P., Robert D. (2013). Modified TiO_2_ for Environmental Photocatalytic Applications: A Review. Ind. Eng. Chem. Res..

[10] Kisch H. (2013). Semiconductor Photocatalysis-Mechanistic and Synthetic Aspects. Angew. Chem.-Int. Ed..

[11] Marschall R. (2014). Semiconductor Composites: Strategies for Enhancing Charge Carrier Separation to Improve Photocatalytic Activity. Adv. Funct. Mater..

[12] Sudha D., Sivakumar P. (2015). Review on the Photocatalytic Activity of Various Composite Catalysts. Chem. Eng. Process. Process Intensif..

[13] Zhu S., Wang D. (2017). Photocatalysis: Basic Principles, Diverse Forms of Implementations and Emerging Scientific Opportunities. Adv. Energy Mater..

[14] Radecka M., Kusior A., Trenczek-Zajac A., Zakrzewska K. (2018). Oxide Nanomaterials for Photoelectrochemical Hydrogen Energy Sources.

[15] Mishra M., Chun D.M. (2015). α-Fe_2_O_3_ as a Photocatalytic Material: A Review. Appl. Catal. A Gen..

[16] Henderson M.A. (2011). A Surface Science Perspective on TiO_2_ Photocatalysis. Surf. Sci. Rep..

[17] Friedmann D., Mendive C., Bahnemann D. (2010). TiO_2_ for Water Treatment: Parameters Affecting the Kinetics and Mechanisms of Photocatalysis. Appl. Catal. B.

[18] Devi L.G., Kavitha R. (2013). A Review on Non Metal Ion Doped Titania for the Photocatalytic Degradation of Organic Pollutants under UV/Solar Light: Role of Photogenerated Charge Carrier Dynamics in Enhancing the Activity. Appl. Catal. B.

[19] Yagub M.T.M.T., Sen T.K.T.K., Afroze S., Ang H.M.M. (2014). Dye and Its Removal from Aqueous Solution by Adsorption: A Review. Adv. Colloid. Interface Sci..

[20] Salleh M.A.M., Mahmoud D.K., Karim W.A.W.A., Idris A. (2011). Cationic and Anionic Dye Adsorption by Agricultural Solid Wastes: A Comprehensive Review. Desalination.

[21] Nosaka Y., Nosaka A.Y. (2017). Generation and Detection of Reactive Oxygen Species in Photocatalysis. Chem. Rev..

[22] Banerjee S., Pillai S.C., Falaras P., O’shea K.E., Byrne J.A., Dionysiou D.D. (2014). New Insights into the Mechanism of Visible Light Photocatalysis. J. Phys. Chem. Lett..

[23] Kusior A., Michalec K., Jelen P., Radecka M. (2019). Shaped Fe_2_O_3_ Nanoparticles–Synthesis and Enhanced Photocatalytic Degradation towards RhB. Appl. Surf. Sci..

[24] Kusior A., Banas J., Trenczek-Zajac A., Zubrzycka P., Micek-Ilnicka A., Radecka M. (2018). Structural Properties of TiO_2_ Nanomaterials. J. Mol. Struct..

[25] Cambier P. (1986). Infrared Study of Goethites of Varying Crystallinity and Particle Size: I. Interpretation of OH and Lattice Vibration Frequencies. Clay Miner..

[26] Cambier P. (1986). Infrared Study of Goethites of Varying Crystallinity and Particle Size: II. Crystallographic and Morphological Changes in Series of Synthetic Goethites. Clay Miner..

[27] Stanjek H., Schwertmann U.D.O. (1992). The Inluence of Alulinum on Iron Oxides, Part XVI: Hydroxyl and Aluminum Substitution on Synthethic Hematites. Clays Clay Miner..

[28] Schwertmann U., Wolska E. (1990). The Influence of Aluminum on Iron Oxides. XV. Al-for-Fe Substitution in Synthetic Lepidocrocite. Clays Clay Miner..

[29] Russell J.D., Paterson E., Fraser A.R., Farmer V.C. (1975). Adsorption of Carbon Dioxide on Goethite (α-FeOOH) Surfaces, and Its Implications for Anion Adsorption. J. Chem. Soc. Faraday Trans. 1 Phys. Chem. Condens. Phases.

[30] Amami B., Addou M., Monty C. (2001). Selfdiffusion and Point Defects in Iron Oxides: FeO, Fe_3_O_4_, α-Fe_2_O_3_. Defect. Diffus. Forum.

[31] Reboiras M.D., Kaszuba M., Connah M.T., Jones M.N. (2001). Measurement of Wall Zeta Potentials and Their Time-Dependent Changes Due to Adsorption Processes: Liposome Adsorption on Glass. Langmuir.

[32] Cornell R.M., Schwertmann U. (2003). The Iron Oxides. Structure, Properties, Reactions, Occurrences and Uses.

[33] Kusior A., Trenczek-Zajac A., Mazurków J., Michalec K., Synowiec M., Radecka M. (2022). Interface Design, Surface-Related Properties, and Their Role in Interfacial Electron Transfer. Part I: Materials-Related Topics. Advances in Inorganic Chemistry.

[34] Lagergren S. (1898). About the Theory of So-Called Adsorption of Soluble Substances. K. Sven. Vetenskapsakademiens Handl..

[35] Doǧan M., Özdemir Y., Alkan M. (2007). Adsorption Kinetics and Mechanism of Cationic Methyl Violet and Methylene Blue Dyes onto Sepiolite. Dye. Pigment..

[36] Yao C., Chen T. (2017). A Film-Diffusion-Based Adsorption Kinetic Equation and Its Application. Chem. Eng. Res. Des..

[37] Wang S., Li H. (2007). Kinetic Modelling and Mechanism of Dye Adsorption on Unburned Carbon. Dye. Pigment..

[38] Srivastava V.C., Swamy M.M., Mall I.D., Prasad B., Mishra I.M. (2006). Adsorptive Removal of Phenol by Bagasse Fly Ash and Activated Carbon: Equilibrium, Kinetics and Thermodynamics. Colloids Surf. A Physicochem. Eng. Asp..

[39] Fondell M., Jacobsson T.J., Boman M., Edvinsson T. (2014). Optical Quantum Confinement in Low Dimensional Hematite. J. Mater. Chem. A Mater..

[40] Butler M.A., Ginley D.S. (1987). Prediction of Flatband Potentials at Semiconductor-Electrolyte Interfaces from Atomic. J. Electrochem. Soc..

[41] Zhang L., Jaroniec M. (2018). Toward Designing Semiconductor-Semiconductor Heterojunctions for Photocatalytic Applications. Appl. Surf. Sci..

[42] Kissling G.P., Ruhstaller B., Pernstich K.P. (2023). Measuring Frontier Orbital Energy Levels of OLED Materials Using Cyclic Voltammetry in Solution. Org. Electron..

[43] Chiu Y.H., Chang T.F.M., Chen C.Y., Sone M., Hsu Y.J. (2019). Mechanistic Insights into Photodegradation of Organic Dyes Using Heterostructure Photocatalysts. Catalysts.

[44] Yang S.J., Lin Y.K., Pu Y.C., Hsu Y.J. (2022). Crystal Facet Dependent Energy Band Structures of Polyhedral Cu_2_O Nanocrystals and Their Application in Solar Fuel Production. J. Phys. Chem. Lett..

